# Synthesis of Amorphous Cellulose Derivatives via Michael Addition to Hydroxyalkyl Acrylates for Thermoplastic Film Applications

**DOI:** 10.3390/polym16223142

**Published:** 2024-11-11

**Authors:** Hiroyuki Nagaishi, Masayasu Totani, Jun-ichi Kadokawa

**Affiliations:** Graduate School of Science and Engineering, Kagoshima University, 1-21-40 Korimoto, Kagoshima 890-0065, Japan; k4784613@kadai.jp (H.N.); m-totani@cb.kagoshima-u.ac.jp (M.T.)

**Keywords:** amorphous derivative, cellulose, hydroxyalkyl acrylate, ionic liquid, Michael addition, thermoplasticity

## Abstract

The aim of this study is to prepare new cellulose derivatives that show good feasibility and processability. Accordingly, in this study, we demonstrate Michael addition to hydroxyalkyl acrylates, that is, 2-hydroxyethyl and 4-hydroxybutyl acrylates (HEA and HBA, respectively), to synthesize amorphous cellulose derivatives under alkaline conditions. The reactions were carried out in the presence of LiOH in ionic liquid (1-butyl-2,3-dimethylimidazolium chloride)/*N*,*N*-dimethylformamide (DMF) solvents at room temperature or 50 °C for 1 h. The Fourier transform infrared and ^1^H nuclear magnetic resonance (NMR) measurements of the products supported the progress of Michael addition; however, the degrees of substitution (DS) were not high (0.3–0.6 for HEA and 0.6 for HBA). The powder X-ray diffraction analysis of the products indicated their amorphous nature. The cellulosic Michael adduct from HEA with DS = 0.6 was swollen with high polar organic liquids, such as DMF. In addition to swelling with these liquids, the cellulosic Michael adduct from HBA was soluble in dimethyl sulfoxide (DMSO), leading to its ^1^H NMR analysis in DMSO-*d*_6_. This adduct was found to form a cast film with flexible properties from its DMSO solutions. Furthermore, films containing an ionic liquid, 1-butyl-3-methylimidazolium chloride, showed thermoplasticity. The Michael addition approach to hydroxyalkyl acrylates is quite effective to totally reduce crystallinity, leading to good feasibility and processability in cellulosic materials, even with low DS. In addition, the present thermoplastic films will be applied in practical, bio-based, and eco-friendly fields.

## 1. Introduction

**Figure 1:**
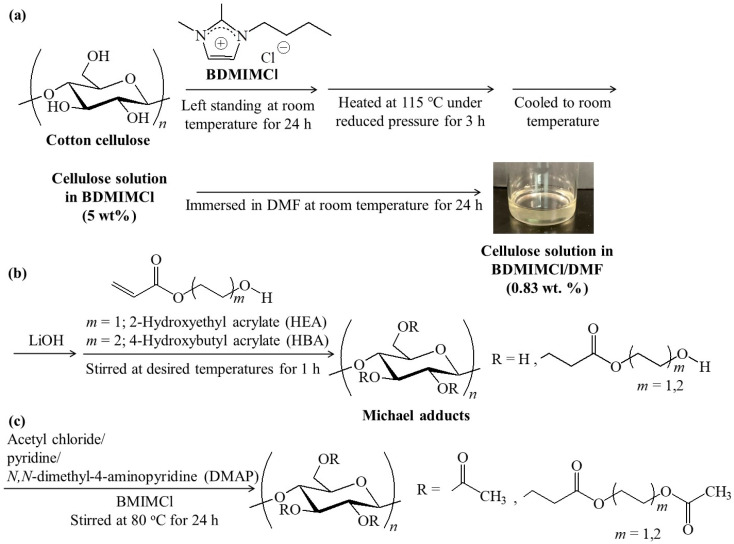
(**a**) Preparation of cellulose solution in 1-butyl-2,3-dimethylimidazoium chloride (BDMIMCl)/*N*,*N*-dimethylformamide (DMF), (**b**) Michael addition to 2-hydroxyethyl or 4-hydroxybutyl acrylate (HEA/HBA) in the presence of LiOH in the solution, and (**c**) acetylation of produced Michael adducts in 1-butyl-3-methylimidazoium chloride (BMIMCl).

Cellulose, which is composed of repeating β(1γ4)-linked glucose units (Figure 1a), is one of the most abundant organic resources on the earth and used as a macromolecular ingredient in practical applications, such as the paper, cloth, and furniture industries [1,2]. However, due to its fibrous and stiff molecular packing, constructed from the cellulosic chains by numerous intra- and intermolecular hydrogen bonds, cellulose shows limitations in the extension of its fields of application. To overcome this drawback, experimental derivatives of cellulose have been produced in order to achieve cellulosic materials with better feasibility and processability compared to those of pure cellulose. Acylation (ester derivatization) and etherification of hydroxy groups in cellulose have been widely investigated using acyl chlorides/acid anhydrides and alkyl halides, respectively [3,4,5,6]. For example, cellulose triacetate (CTA) is one of the most well-known, which has been produced by acetylation of cellulose using acetyl chloride or acetic anhydride under appropriate conditions. CTA is commercially used as a practical, polymeric material for applications such as optical compensation film for liquid crystal display, protective film for polarizing plates, and photographic film, owing to its thermoplasticity [3]. However, CTA is nearly not melt-processable due to its high melting point. Cellulose ester derivatives with longer acyl substituents, such as cellulose tripropionate, show better processability than CTA owing to their lower melting and glass transition temperatures [4]. In cellulose ether derivatives, on the other hand, hydroxypropyl cellulose, which is prepared by a ring-opening reaction of propylene oxide on cellulose in aqueous NaOH, is practically employed for transdermal patches, ophthalmic lubricants, and drug encapsulants [5]. The facts above indicate that cellulosic derivatives exhibit their intrinsic nature and properties depending on the kinds of substituents. Accordingly, development of new derivatization methods for cellulose efficiently leads to the fabrication of new, bio-based functional materials for practical applications, such as in biomedical, eco-friendly, and environmentally benign fields [6,7,8,9,10].

As specific derivatizations of cellulose other than common derivatizations, such as the above acylation and etherification, Michael addition using acrylamide from cellulose was investigated [11,12,13]. Michael addition is the reaction for new bond formation through nucleophilic attack of appropriate nucleophiles to α,β-unsaturated carbonyl compounds as electrophiles. Because Michael addition has served important roles in polymer synthesis, allowing the development of novel polymers [14], it can also be employed for derivatization reactions of polymeric substrates. For example, during the above approach via Michael addition for synthesis of new polymeric materials from cellulose, alkoxides, generated from hydroxy groups in cellulose under alkaline conditions, add to acrylamide to produce a cellulosic Michael adduct derivative. Alkaline hydrolysis of amido linkages in the produced derivative was then conducted to obtain a carboxyethyl ether derivative called cellulose acrylate. The cellulose acrylate showed water solubility and low crystallinity, despite its low degree of substitution (DS). Furthermore, flocculation–coagulation behavior of this derivative was investigated, leading to its application to the removal of Cu (II) ions and colloidal Fe(OH)_3_ turbidity.

On the other hand, ionic liquids have attracted much attention as solvents for cellulose [15,16,17,18,19,20,21,22,23,24,25,26] since, in 2002, 1-butyl-3-methylimidazoium chloride (BMIMCl) was found to dissolve cellulose [27]. We have found a facile procedure for production of homogeneous cellulosic organic solutions by immersing 5 wt.% cellulose/BMIMCl solutions in organic liquids with high polarities, such as *N*,*N*-dimethylformamide (DMF) and *N*-methyl-2-pyrrolidone (NMP) [28]. In the following study, we also achieved ring-opening graft polymerization of 1,2-butylene oxide from cellulose in the presence of NaH in the resulting cellulose/BMIMCl/NMP solution [29].

In the present study, we investigated the Michael addition of cellulose to the other α,β-unsaturated carbonyl compounds as electrophiles, that is, 2-hydroxyethyl and 4-hydroxybutyl acrylates (HEA and HBA, respectively) under alkaline conditions in homogeneous cellulose/ionic liquid/DMF solutions, prepared by the abovementioned procedure (Figure 1a,b). As the ionic liquid, we used 1-butyl-2,3-dimethylimidazolium chloride (BDMIMCl), which was also reported to dissolve cellulose, in place of BMIMCl [30]. Because of the presence of an acidic hydrogen at position 2 on the imidazolium in BMIMCl [31], which has the potential to prevent Michael addition from alkoxides on cellulose to the electrophiles under strong alkaline conditions, the hydrogen at position 2 has been substituted with a methyl group on the imidazolium in BDMIMCl. The produced cellulosic Michael adducts showed a mostly amorphous nature, although their DS values were not high. Dissolution and swelling behaviors of the cellulosic Michael adducts with high polar organic liquids were investigated in accordance with the two hydroxyalkyl acrylates and DS values. Furthermore, acetylation of the Michael adducts was performed to further confirm the structures of the substituents (Figure 1c). We also found that cast films were formed from the cellulosic Michael adducts using HBA, which showed thermoplasticity by incorporating BMIMCl as a plasticizer. Based on the viewpoint of feasibility (solubility and swellability) and processability (thermoplasticity), accordingly, the present Michael adducts with quite low DS can be considered to be superior to some of the other cellulose derivatives for future practical applications [32]. Moreover, the Michael addition approach can extend to different α,β-unsaturated carbonyl compounds to obtain new cellulose derivatives.

## 2. Materials and Methods

### 2.1. Materials

Absorbent cotton with average degrees of polymerization of 2000–5000 was purchased from Hakujuji Corporation, Tokyo, Japan. The ionic liquid BDMIMCl was synthesized via quaternization of 1,2-dimethylimidazole (>98%, Tokyo Chemical Industry Co., Ltd., Tokyo, Japan) with 1-chlorobutane (>98%, FUJIFILM Wako Pure Chemical Corporation, Osaka, Japan) according to the method adapted from the procedure in [30]. The ionic liquid BMIMCl was synthesized via quaternization of 1-methylimidazole (>99%, Tokyo Chemical Industry Co., Ltd., Tokyo, Japan) with 1-chlorobutane (>98%, FUJIFILM Wako Pure Chemical Corporation, Osaka, Japan) according to the method adapted from the procedure in [33]. DMF (>99.5%), LiOH (100%), acetyl chloride (>98%), pyridine (>99.5%), and *N*,*N*-dimethyl-4-aminopyridine (DMAP, >99%) were purchased from FUJIFILM Wako Pure Chemical Corporation, Osaka, Japan. HEA (>95%) and HBA (>97%) were purchased from Tokyo Chemical Industry Co., Ltd., Tokyo, Japan. Commercially available high-polar organic liquids were used as received.

### 2.2. Preparation of Cellulose Solution in BDMIMCl/DMF

A mixture of cotton cellulose (0.040 g, 0.25 mmol) with BDMIMCl (0.75 g, 5.70 mmol) was left standing at room temperature for 24 h and subsequently heated at 115 °C for 3 h under reduced pressure to obtain a 5 wt.% cellulose solution. The resulting solution was immersed in DMF (4.0 mL) for 24 h under ambient atmosphere to form a homogeneous solution (0.83 wt.%).

### 2.3. Michael Addtion of Cellulose to Hydroxylalkyl Acrylates in BDMIMCl/DMF Solution

A typical experimental procedure was as follows (run 3 in Table 1). Under argon, LiOH (0.060 g, 2.50 mmol, 3.3 equiv. with hydroxy groups in cellulose) was added to the abovementioned solution at room temperature with stirring, and the mixture was maintained for 1 h. After HBA (2.0 mL, 14.8 mmol, 20.0 equiv. with hydroxy groups in cellulose) was added to the mixture, it was stirred at room temperature for 1 h. After the reaction mixture was neutralized with 0.10 mol/L hydrochloric acid (ca. 6 mL), the precipitated material was isolated by centrifugation, washed with acetone (30 mL) and water (30 mL), and lyophilized to give the cellulosic Michael adduct (0.037 g). ^1^H nuclear magnetic resonance (NMR, NaOD/D_2_O) δ 1.55–1.64 (br, -C-CH_2_CH_2_-C-), 2.43–2.52 (br, -CH_2_C=O), 3.25–4.12 (br m, -CH_2_O-, H2-H6 of glucose), 4.46–4.56 (br, H1 of glucose). ^1^H NMR (DMSO-*d*_6_) δ 1.39–1.71 (br m, -C-CH_2_CH_2_-C-), 2.44–2.55 (br, -CH_2_C=O, overlapping with solvent (DMSO) signal), 2.98–3.83 (br m, -C*H*_2_OH, H2-H6 of glucose, partly overlapping with moisture (HOD) signal), 3.95–4.06 (br, CH_2_OC=O), 4.20–4.36 (br, H1 of glucose), 4.55–4.80, 5.30–5.49 (br, OH).

The same experimental procedures were also conducted for runs 1, 2, and 4 (Table 1) at desired temperatures.

### 2.4. Acetylation of Cellulosic Michael Adducts

A typical experimental procedure was as follows (run 3). The cellulosic Michael adduct (0.020 g) was dissolved in BMIMCl (2.0 g) by heating a mixture at 80 °C for 24 h. After acetyl chloride (0.442 mL, 5.64 mmol), pyridine (0.178 mL, 2.2 mL), and DMAP (4.0 mg, 0.0245 mmol) were added to the solution, the resulting mixture was heated at 80 °C for 24 h with stirring. Methanol (30 mL) was then added to the reaction mixture, followed by ultrasonication for 10 min, to precipitate the acetylated derivative, which was isolated by centrifugation, and lyophilized (0.0232 g). ^1^H NMR (CDCl_3_) δ 1.65–1.77 (br, -C-CH_2_CH_2_-C-), 1.95, 2.02, 2.13 (s, CH_3_C=O of peracetyl glucose), 2.06 (s, CH_3_C=O of substituent terminus), 2.49–2.62 (br, -CH_2_C=O), 3.49–3.59 (br, H5 of glucose), 3.64–3.90 (br, H4 of glucose), 4.00–4.14 (br, H6_a_ of glucose), 4.28–4.55 (br, H6_b_ and H1 of glucose), 4.71–4.95 (br, H2 of glucose), 4.95–5.20 (br, H3 of glucose).

### 2.5. Film Formation and Evaluation of Thermiplasticity of Michael Adducts

Solutions of the cellulosic Michael adducts and 0–2 wt.% of BMIMCl (total 0.150 g) in DMSO (2.0 mL) were casted on a silicone rubber cup and dried at 60 °C for 12 h to obtain films. Two pieces of the film were partly layered and pressed at 1 MPa at 180 °C for 10 min for evaluation of thermoplasticity.

### 2.6. Measurements

Fourier transform infrared (FTIR) spectra were recorded on a PerkinElmer Spectrum Two spectrometer (PerkinElmer Japan Co., Ltd., Yokohama, Japan). ^1^H NMR spectra were recorded using a JEOL ECX400 spectrometer (JEOL, Akishima, Tokyo, Japan). Powder X-ray diffraction (XRD) measurements were performed using a PANalytical X’Pert Pro MPD instrument (PANalytical B.V., Almelo, The Netherlands) with Ni-filtered Cu-Kα radiation (λ = 0.15418 nm). Scanning electron microscopic (SEM) images were obtained using a Hitachi SU-70 electron microscope (Hitachi High-Technologies Corporation, Tokyo, Japan) at an accelerating voltage of 5 kV. The stress–strain curves were measured using a tensile tester (Little Senstar LSC-1/30, Tokyo Testing Machine, Tokyo Japan). Differential scanning calorimetry (DSC) was performed using a DSC 6220 calorimeter (Seiko Instruments Inc. Chiba, Japan).

## 3. Results and Discussion

Prior to conducting Michael addition of cellulose to HEA/HBA, a homogeneous cotton cellulose solution in BDMIMCl/DMF (0.83 wt.%) was prepared according to the previous procedure reported by us (see Section 2.2, Figure 1a) [28]. After the cellulose in the obtained solution was treated with LiOH (3.3 equiv. with hydroxy groups in cellulose) with stirring to generate alkoxides as nucleophiles from its hydroxy groups, HEA or HBA (20 equiv. with hydroxy groups in cellulose) was added to the mixture. The resulting mixture was then maintained at room temperature or 50 °C for 1 h with stirring for the progress of Michael addition (Figure 1b). The reaction mixture was neutralized with 0.10 mol/L hydrochloric acid; the precipitate obtained in the mixture was isolated by centrifugation, washed with acetone and water, and lyophilized. The products were subjected to the following structural characterization by FTIR and ^1^H NMR measurements.

**Figure 2:**
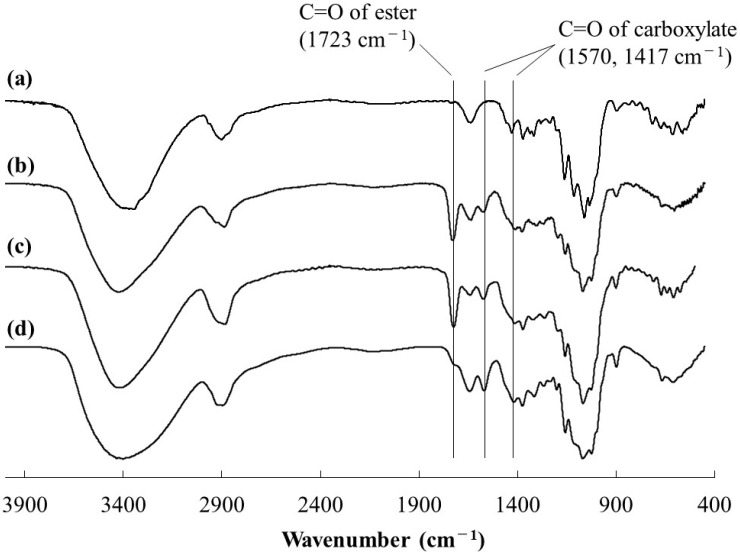
FTIR spectra of (**a**) cellulose, (**b**) cellulosic Michael adduct from HEA (run 1), (**c**) cellulosic Michael adduct from HBA (run 3), and (**d**) dried material from alkaline hydrolysate (run 3).

**Figure 3:**
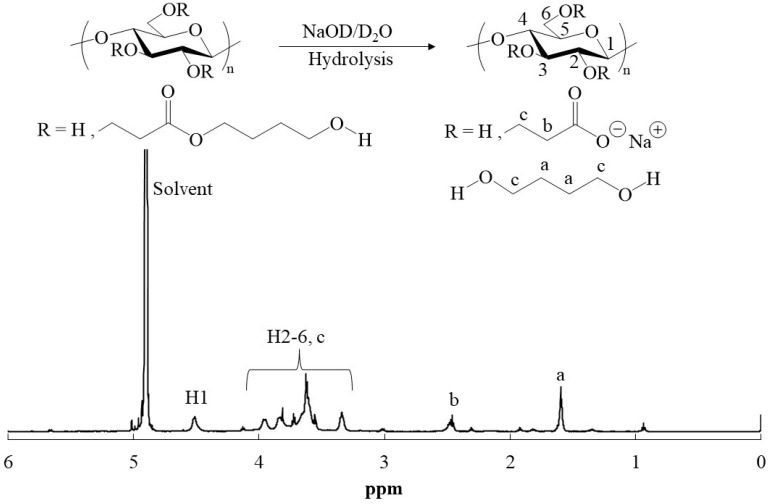
^1^H NMR spectrum of hydrolysate of cellulosic Michael adduct from HBA (run 3) in NaOD/D_2_O.

Compared to the FTIR spectrum of cellulose (Figure 2a), the FTIR spectra of all products (runs 1–4) had newly exhibited carbonyl absorptions at 1723 cm^−1^ assignable to ester linkage, as the representative spectra of the products from HEA (run 1) and HBA (run 3) are shown in Figure 2b,c, respectively. These results indicate incorporation of ester moieties in the cellulosic products by Michael addition from alkoxides to HEA/HBA. For the further characterization by ^1^H NMR measurement, the products were solubilized in aqueous solvents by alkaline hydrolysis of the ester linkages in the substituents of NaOD/D_2_O. The ^1^H NMR spectrum of the hydrolysate from the product from HEA (run 1) showed not only signals at δ 3.23–4.01 (H2-H6) and 4.44–4.55 (H1) derived from glucose units, but also a signal at δ 2.43–2.48 ascribed to the methylene protons adjacent to a carbonyl group (-CH_2_C=O) (Figure S1). The detection of the latter signal suggests the occurrence of Michael addition to incorporate the -OCH_2_CH_2_(C=O)O- substituents on cellulose. The signals assigned to the oxymethylene protons (-OCH_2_-) in such substituents as well as the signals derived from 1,2-ethanediol, produced by alkaline hydrolysis of the substituent (-OCH_2_CH_2_(C=O)OCH_2_CH_2_OH), were notably overlapped with glucose signals in the spectrum. The ^1^H NMR spectrum of the hydrolysate from the product from HBA (run 3) showed a signal at δ 1.55–1.64, in addition to the same signals as those in the above spectrum, which was assignable to the methylene protons (-CCH_2_CH_2_C-) in 1,4-butanediol, produced by alkaline hydrolysis of the substituent (-OCH_2_CH_2_(C=O)OCH_2_CH_2_CH_2_CH_2_OH) (Figure 3).

The FTIR spectra of the dried material from the alkaline hydrolysate (run 3) exhibited absorptions ascribed to carboxylate groups at 1570 and 1417 cm^−1^ derived from -OCH_2_CH_2_(C=O)ONa, which were formed by alkaline hydrolysis of the substituents (Figure 2d). The presence of the carboxylate groups on cellulose chains was considered to contribute to solubilization of the hydrolysates from the products in aqueous media for the above NMR analysis. Accordingly, the ^1^H NMR spectroscopic patterns of the alkaline hydrolysates (runs 1 and 3) in Figure S1 and Figure 3 were identical with those in the ^1^H NMR spectra of the abovementioned cellulose acrylate, reported in the previous studies [11,13], because of the presence of the same substituents (-OCH_2_CH_2_(C=O)ONa) on cellulose. All the above characterization results strongly supported that Michael addition from alkoxides on cellulose to HEA/HBA in BDMIMCl/DMF solvents occurred to obtain the cellulosic derivatives (cellulosic Michael adducts) having the -CH_2_CH_2_(C=O)O(CH_2_CH_2_)*_m_*OH substituents.

The DS values of the substituents were calculated from the integrated ratios of the -CH_2_C=O signals in the substituents to the H1 signals in the glucose units in the ^1^H NMR spectra of the hydrolysates in NaOD/D_2_O, as shown in Table 1. When HEA was used, the DS values increased from 0.3 to 0.6 by elevating the reaction temperatures (room temperature γ 50 °C). On the other hand, the DS values remained intact (0.3) regardless of the reaction temperatures in the case of using HBA.

**Figure 4:**
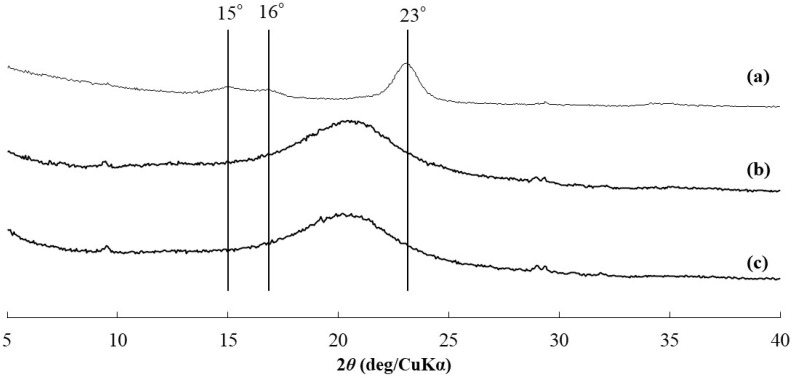
XRD profiles of (**a**) cellulose, (**b**) cellulosic Michael adduct from HEA (run 1), and (**c**) cellulosic Michael adduct from HBA (run 3).

The XRD profiles of all the cellulosic Michael adducts (representatively shown in Figure 4b,c for runs 1 and 3) did not show obvious diffraction peaks at 15, 16, and 23° assignable to cellulose crystalline structure (cellulose I), as detected in that of the original cotton cellulose (Figure 4a). These results indicate that cellulose chains did not construct controlled alignments in the cellulosic Michael adducts, probably owing to the prevention of regular hydrogen bonding, by introducing the -CH_2_CH_2_(C=O)O(CH_2_CH_2_)*_m_*OH substituents even with low DS.

**Figure 5:**
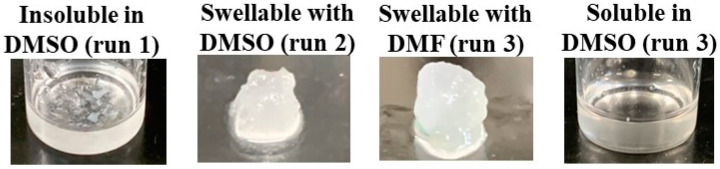
Photographs of mixtures of Michael adducts with DMSO and DMF after shaking at room temperature.

**Figure 6:**
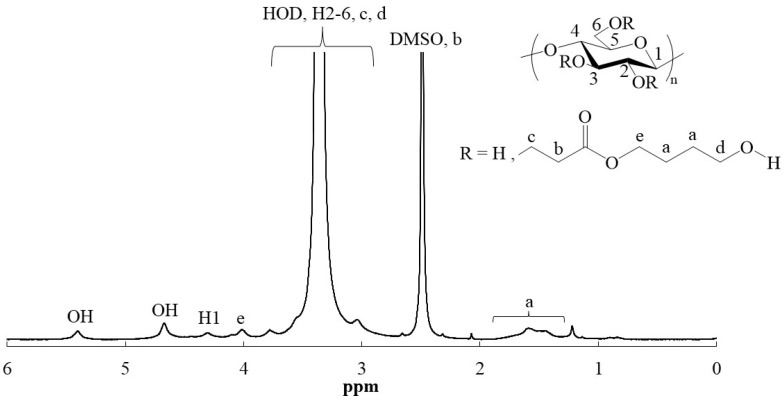
^1^H NMR spectrum of cellulosic Michael adduct from HBA (run 3) in DMSO-*d*_6_.

The behavior of the present cellulosic Michael adducts toward several high-polar organic liquids was then explored (Table 2). Each cellulosic Michael adduct (5.0 mg) was mixed with 1.0 mL of respective liquids, and the mixtures were shaken at room temperature. The cellulosic Michael adduct of run 1 (from HEA, DS = 0.3) was not soluble and swollen in any high-polar organic liquids tested (dimethyl sulfoxide (DMSO), DMF, *N*,*N*-dimethylacetamide (DMAc), NMP, and 1,2-ethanediol), as representatively shown in Figure 5. The cellulosic Michael adduct of run 2 with the higher DS (0.6), obtained from HEA, was swollen with all liquids (Figure 5). On the other hand, the cellulosic Michael adducts of runs 3 and 4, obtained from HBA, were swollen with NMP, DMAc, DMF, and 1,2-ethanediol, despite their low DS (0.3, Figure 5). Interestingly, as these cellulosic Michael adducts were found to be soluble in DMSO, as shown in Figure 5, their ^1^H NMR analysis in DMSO-*d*_6_ was conducted. The ^1^H NMR spectrum of the Michael adduct of run 3 in DMSO-*d*_6_ showed the signals derived from the glucose units and the -CH_2_CH_2_(C=O)OCH_2_CH_2_CH_2_CH_2_OH substituents, including the methylene signal assignable to alkyl ester (O=COCH_2_-) at δ 3.95–4.06 (Figure 6). The methylene signal was not detected in the ^1^H NMR spectrum of the hydrolysate from the cellulosic Michael adduct of run 3 in NaOD/D_2_O (Figure 3) because of the absence of ester groups by their alkaline hydrolysis.

**Figure 7:**
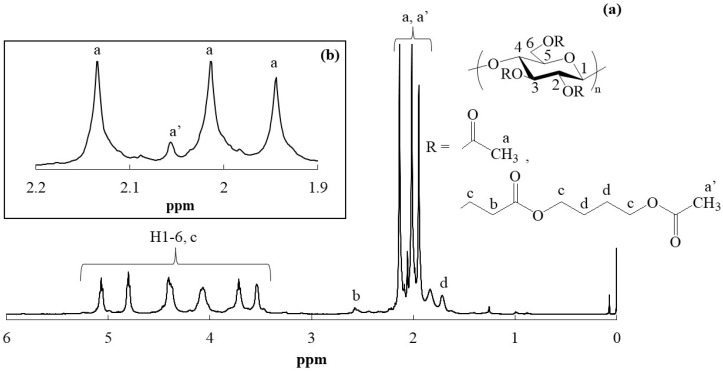
(**a**) ^1^H NMR spectrum of acetylated derivative, prepared from cellulosic Michael adduct of run 3 in CDCl_3_ and (**b**) expanded region for acetyl methyl signals.

Hydroxyalkyl acrylates are known to polymerize each other via consecutive Michael additions under alkaline conditions [34]. To confirm whether such polymerization of HEA/HBA from the end groups in the -CH_2_CH_2_(C=O)OCH_2_CH_2_CH_2_CH_2_OH substituents occurred during the present reaction systems, acetylation of the cellulosic Michael adducts of runs 1 and 3 was examined (Figure 1c). After dissolution of the cellulosic Michael adducts in BMIMCl, the acetylation was carried out using acetyl chloride in the presence of pyridine/DMAP as base and catalyst, respectively, at 80 °C for 24 h. The ^1^H NMR spectra of the acetylated products, isolated as fractions insoluble in methanol, in CDCl_3_ exhibited the individual H1-H6 signals ascribed to acetylated glucose units and the three methyl signals assigned to CH_3_C=O groups (Figure S2a and Figure 7a, signals’ assignments are shown in Section 2.4), suggesting that the acetylation was fully progressed under the above conditions. Although the spectroscopic patterns were almost identical with those of pure cellulose triacetate [35] because of the quite low DS values (0.3) in the cellulosic Michael adducts, the -CH_2_C=O signals derived from the substituents were detected at around δ 2.6. Furthermore, the methyl signals assignable to the acetate groups, produced by reaction of the terminal hydroxy groups in the substituents with acetyl chloride, were also detected at δ 2.06–2.08 between the abovementioned methyl signals derived from the peracetyl glucose units (expanded spectra in Figure S2b and Figure 7b). The degrees of polymerization for the substituents could be estimated by the integrated ratios of the terminal methyl signals to the -CH_2_C=O signals, which were 1.07 and 1.05 for the acetylated products of runs 1 and 3, respectively. These results indicate that further polymerization of HEA/HBA from the end groups in the -CH_2_CH_2_(C=O)OCH_2_CH_2_CH_2_CH_2_OH substituents via Michael addition did not mostly occur in the present reaction systems.

**Figure 8:**
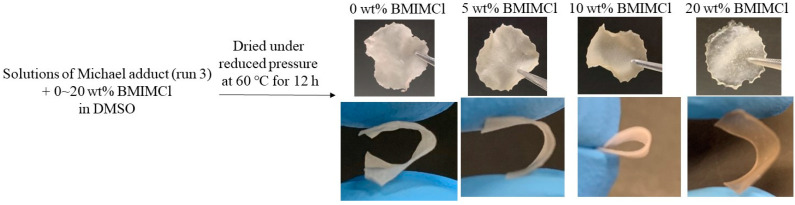
Preparation of cast films from solutions of cellulosic Michael adduct of run 3 containing 0 and 20 wt.% BMIMCl in DMSO and their bending performance.

**Figure 9:**
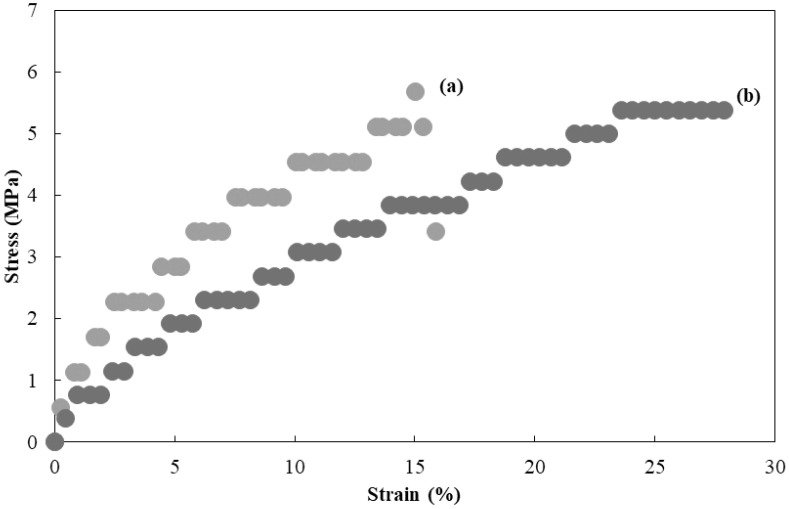
Stress-strain curves of cast films from solutions of cellulosic Michael adduct of run 3 containing 0 and 20 wt.% BMIMCl ((**a**) and (**b**), respectively) under tensile mode.

The cellulosic Michael adduct of run 3 was found to form a cast film by drying of its DMSO solution (Figure 8). Furthermore, because ionic liquids such as BMIMCl were reported to act as plasticizers for cellulosic materials [36,37,38], cast films of the cellulosic Michael adducts with BMIMCl (5–20 wt.%) could also be obtained from the corresponding DMSO solutions (Figure 8). All the films obtained were flexible, as they were bent well, as shown in Figure 8. The surface morphologies of the films were evaluated by SEM measurement. The SEM image of the film containing 20 wt.% BMIMCl was obviously not different from that of the film in the absence of BMIMCl (Figure S3). These results suggest that the addition of BMIMCl did not affect the surface morphologies mainly. The stress–strain curves of the films containing 0 and 20 wt.% BMIMCl resulting from tensile testing (Figure 9) indicate the contribution of BMIMCl to enhancing toughness because values of elongation at break increased by incorporation of BMIMCl (15.3 γ 28.4%, respectively) with maintaining the tensile strength (ca. 5 MPa). These results suggest the role of BMIMCl in the films as a plasticizer.

**Figure 10:**
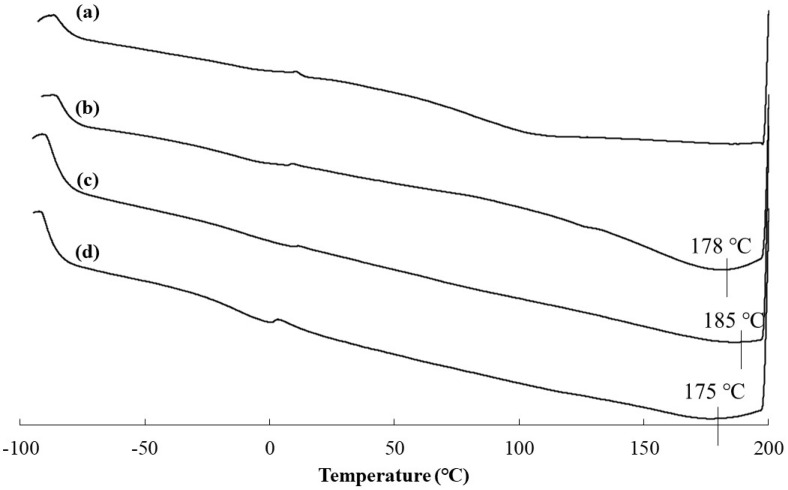
DSC profiles of cast films from solutions of cellulosic Michael adduct of run 3, containing (**a**) 0, (**b**) 5, (**c**) 10, and (**d**) 20 wt.% BMIMCl.

**Figure 11:**
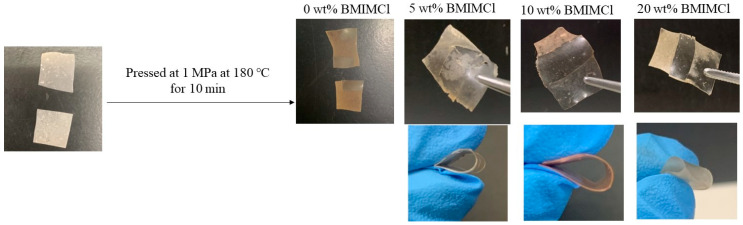
Melt-pressing experiment of cast films from solutions of cellulosic Michael adduct of run 3, containing 0–20 wt.% BMIMCl for evaluation of thermoplasticity and bending performance after melt pressing.

The DSC profiles of the films containing 5–20 wt.% BMIMCl exhibited endothermic peaks at around 180 °C (Figure 10b–d), whereas such a peak was not detected in those of the film with an absence of BMIMCl (Figure 10a). The results suggest that the presence of BMIMCl probably provided thermoplasticity in the films. To evaluate the thermoplasticity, melt-pressing experiments of the films were carried out. When two pieces of the films containing 5–20 wt.% BMIMCl were partially layered and pressed at 1 MPa at 180 °C for 10 min, adhesion of the pieces was observed (Figure 11). The resulting films after melt pressing could be bent similarly to those before melt pressing (Figure 11). On the other hand, such adhesion was not observed by the same operation of the film in the absence of BMIMCl (Figure 11). The results strongly supported the production of thermoplastic materials from the cellulosic Michael adducts combined with BMIMCl as a plasticizer. Furthermore, much smaller amounts of ionic liquids efficiently act as the plasticizer for cellulose derivatives in the films, compared to the previous studies [36,37]. Additional characterization of the films will be investigated in future work.

## 4. Conclusions

In this study, we successfully synthesized the amorphous cellulose derivatives, that is, the cellulosic Michael adducts, by Michael addition of alkoxides on cellulose, generated under alkaline conditions, to HEA and HBA. The progress of Michael addition and the amorphous nature of the products were supported by the FTIR/^1^H NMR and XRD measurements, respectively. In addition to the swellability of the cellulosic Michael adducts with high-polar organic liquids, those from HBA were soluble in DMSO. Casting and drying of the DMSO solution yielded a flexible film, which was also combined with BMIMCl. The films containing BMIMCl exhibited thermoplasticity, supported by the DSC measurement and melt-pressing experiment. This study revealed that the present derivatization via Michael addition to hydroxyalkyl acrylates is a very useful approach to totally reduce crystallinity and provide solubility and swellability of cellulosic materials, even with low DS. The thermoplastic films prepared from the cellulosic Michael adducts from HBA and BMIMCl have the potential to be practically employed as bio-based and eco-friendly materials. Furthermore, in the future, the present Michael addition approach can be applied to the other α,β-unsaturated carbonyl compounds to produce new cellulose derivatives with unique functions.

## Figures and Tables

**Table 1:** Michael addition of cellulose to hydroxyalkyl acrylates in the presence of LiOH for 1 h in BDMIMCl/DMF solvent ^(a)^.

Run	Substrate ^(b)^	Temperature	Yield (mg)	DS ^(c)^
1	HEA	room temperature	41.9	0.3
2	HEA	50 °C	34.4	0.6
3	HBA	room temperature	37.0	0.3
4	HBA	50 °C	41.8	0.3

^(a)^ Reaction was carried out using 40 mg of cellulose. ^(b)^ HEA; 2-hydroxyethyl acrylate, HBA; 4-hydroxybutyl acrylate. ^(c)^ Determined by ^1^H NMR analysis of hydrolysates of cellulosic Michael adducts in NaOD/D_2_O.

**Table 2:** Solubility and swellability of cellulosic Micheal adducts in polar organic liquids ^(a)^.

Run	DMSO	DMF	DMAc	NMP	1,2,-Ethanediol
1	-	-	-	-	-
2	±	±	±	±	±
3	+	±	±	±	±
4	+	±	±	±	±

^(a)^ Each cellulosic Michael adduct (5.0 mg) was mixed with respective liquids (1.0 mL). The mixtures were then shaken at room temperature. ^(b)^ +, soluble; ±, swellable; -, insoluble and non-swellable.

## Data Availability

Data are contained within the article and supplementary materials.

## Supplementary Materials

The following supporting information can be downloaded at https://www.mdpi.com/article/10.3390/polym16223142/s1, Figure S1: ^1^H NMR spectrum of hydrolysate of cellulosic Michael adduct from HEA (run 1) in NaOD/D_2_O; Figure S2: (a) ^1^H NMR spectrum of acetylated derivative, prepared from cellulosic Michael adduct of run 1 in CDCl_3_ and (b) expanded region for acetyl methyl signals; Figure S3: SEM images of cast films from solutions of cellulosic Michael adduct of run 3 containing 0 and 20 wt.% BMIMCl (a and b, respectively).
