# Supplementary material for: Synthesis of Amorphous Cellulose Derivatives via Michael Addition to Hydroxyalkyl Acrylates for Thermoplastic Film Applications
Source: Polymers (Basel). 2024 Nov 11;16(22):3142. doi: 10.3390/polym16223142 (PMC11598606; doi:10.3390/polym16223142)
Supplement: Supplementary file 1 [file polymers-16-03142-s001.zip › polymers-3305008-supplementary.pdf]

Supporting Information

Synthesis of Amorphous Cellulose Derivatives via Michael Addition to Hydroxyalkyl Acrylates for Thermoplastic Film Applications

Hiroyuki Nagaishi [1], Masayasu Totani [1] and Jun-ichi Kadokawa [1,*]

[1] Graduate School of Science and Engineering, Kagoshima University, 1-21-40 Korimoto, Kagoshima 890-0065, Japan; k4784613@kadai.jp (H.N.); m-totani@cb.kagoshima-u.ac.jp (M.T.)

* Corresponding author: E-mail: kadokawa@eng.kagoshima-u.ac.jp

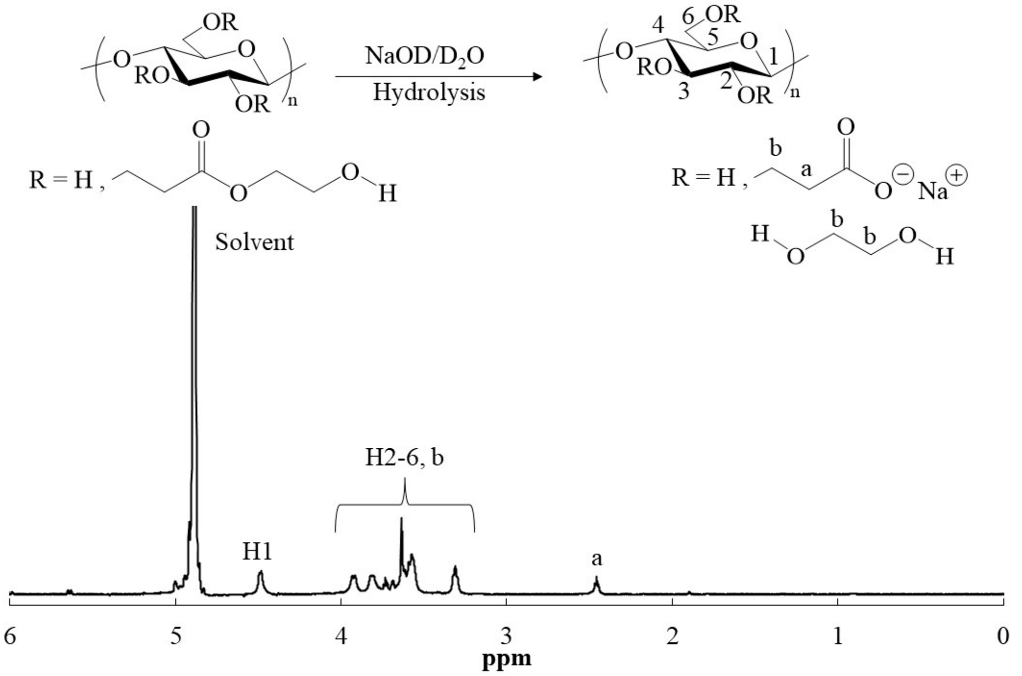

Figure S1. ^{1}H NMR spectrum of hydrolysate of cellulosic Michael adduct from HEA (run 1) in NaOD/D_2O.

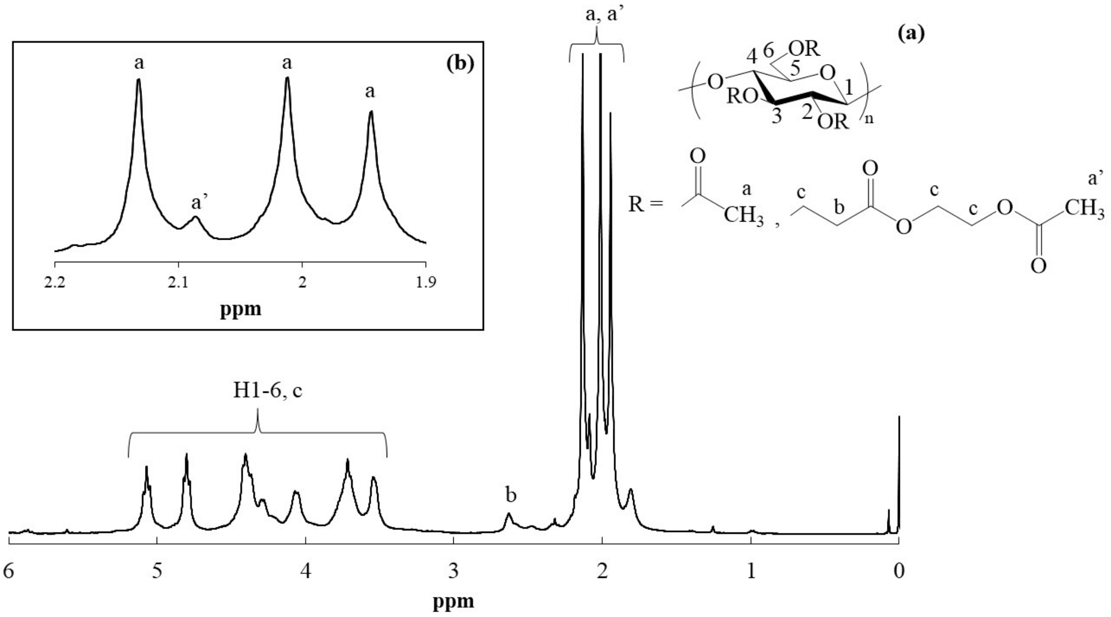

Figure S2. (a) ^{1}H NMR spectrum of acetylated derivative, prepared from cellulosic Michael adduct of run 1 in $CDCl_3$ and (b) expanded region for acetyl methyl signals.

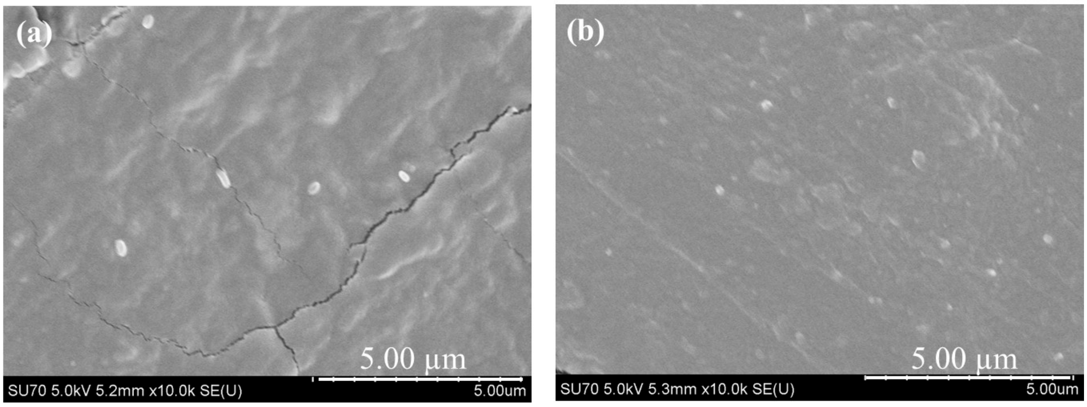

Figure S3. SEM images of cast films from solutions of cellulosic Michael adduct of run 3 containing 0 and 20 wt% BMIMCl (a and b, respectively).
